# Ultra-high-field MRI of postmortem human fetal wrist joints: initial experience

**DOI:** 10.1186/s41747-023-00341-0

**Published:** 2023-06-05

**Authors:** Sabine H. Josemans, Anne-Sophie van der Post, Gustav J. Strijkers, Yousif Dawood, Maurice J. B. van den Hoff, Sjoerd R. J. Jens, Miryam C. Obdeijn, Roelof-Jan Oostra, Mario Maas

**Affiliations:** 1 Department of Radiology and Nuclear Medicine, Amsterdam UMC Location University of Amsterdam, Meibergdreef 9, 1105 AZ Amsterdam, The Netherlands; 2 Sports and Musculoskeletal Health, Amsterdam Movement Sciences, Amsterdam, The Netherlands; 3 Biomedical Engineering and Physics, Amsterdam UMC Location University of Amsterdam, Amsterdam, The Netherlands; 4 Medical Biology, Amsterdam UMC Location University of Amsterdam, Amsterdam, The Netherlands; 5 Radiology and Nuclear Medicine, Rijnstate Hospital, Arnhem, The Netherlands; 6 Plastic, Reconstructive and Hand Surgery, Amsterdam UMC Location University of Amsterdam, Amsterdam, The Netherlands

**Keywords:** Fetus, Magnetic resonance imaging, Musculoskeletal system, Triangular fibrocartilage, Upper extremity

## Abstract

**Background:**

This study aimed to assess the feasibility of postmortem ultra-high-field magnetic resonance imaging (UHF-MRI) to study fetal musculoskeletal anatomy and explore the contribution of variation in iodine and formaldehyde (paraformaldehyde, PFA) treatment of tissue.

**Methods:**

Seven upper extremities from human fetuses with gestational ages of 19 to 24 weeks were included in this experimental study, approved by the Medical Research Ethics Committee. The specimens were treated with various storage (0.2–4% PFA) and staining (Lugol’s solution) protocols and the wrist joint was subsequently imaged with 7.0 T UHF-MRI. Soft-tissue contrast was quantified by determining regions of interest within a chondrified carpal bone (CCB) from the proximal row, the triangular fibrocartilage (TFC), and the pronator quadratus muscle (PQM) and calculating the contrast ratios (CRs) between mean signal intensities of CCB to TFC and CCB to PQM.

**Results:**

UHF-MRI showed excellent soft-tissue contrast in different musculoskeletal tissues. Increasing storage time in 4% PFA, CRs decreased, resulting in a shift from relatively hyperintense to hypointense identification of the CCB. Storage in 0.2% PFA barely influenced the CRs over time. Lugol’s solution caused an increase in CRs and might have even contributed to the inversion of the CRs.

**Conclusions:**

UHF-MRI is a feasible technique to image musculoskeletal structures in fetal upper extremities and most successful after short storage in 4% PFA or prolonged storage in 0.2% PFA. The use of Lugol’s solution is not detrimental on soft-tissue MRI contrast and therefore enables effectively combining UHF-MRI with contrast-enhanced micro-computed tomography using a single preparation of the specimen.

**Relevance statement:**

UHF-MRI can be performed after CE-micro-CT to take advantage of both techniques.

**Key points:**

• UHF-MRI is feasible to study human fetal cartilaginous and ligamentous anatomy.

• Storage in low PFA concentrations (*i.e.*, 0.2%) improves soft-tissue contrast in UHF-MRI.

• Limited preservation time in high concentrations of PFA improves soft-tissue contrast in UHF-MRI.

• Prior staining with Lugol’s solution does not reduce soft-tissue contrast in UHF-MRI.

**Graphical Abstract:**

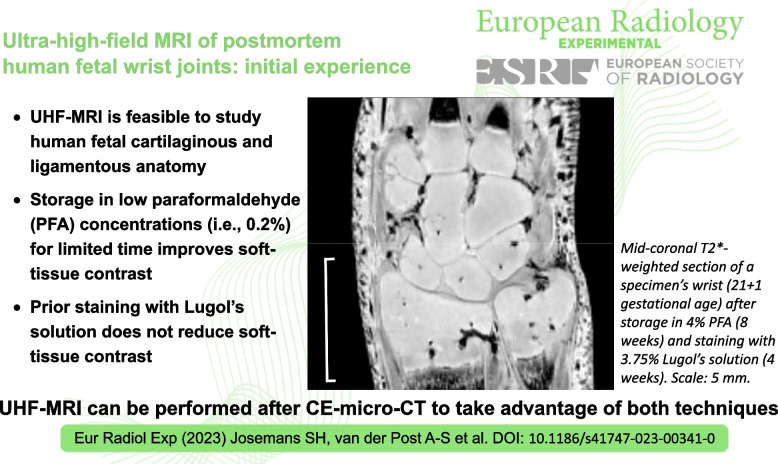

## Background

Knowledge regarding human fetal three-dimensional (3D) musculoskeletal development was generally based on two-dimensional (2D) histological studies [1–3]. Nowadays, the need for direct comparison of histopathology with 3D imaging is apparent in studies on embryological development assuring accurate anatomical topography [4, 5]. In this respect, postmortem high-resolution micro-computed tomography (micro-CT) and ultra-high field MRI (UHF-MRI) have recently received considerable attention [6–9].

Visualization of fetal musculoskeletal structures appears insufficient using micro-CT due to the lack of contrast between cartilage and other soft tissues [10]. To overcome this issue and generate soft-tissue contrast using micro-CT, iodine-based Lugol’s solution is currently most frequently used as a contrast agent [6]. This technique has several drawbacks, such as the labor-intensive process, visible tissue coloration, and tissue shrinkage, although the latter problem has recently been substantially reduced by stabilizing the pH using a buffered Lugol’s solution since a decrease in pH level induced tissue shrinkage [11–13].

On the other hand, MRI with fast-spin-echo and gradient-echo sequences is known for its excellent soft-tissue contrast, and therefore generally the modality of choice in clinical musculoskeletal imaging [14–16]. Postmortem UHF-MRI, generally performed using MRI with a magnetic field strength $\ge$ 7 T, has been shown to provide high-resolution imaging of early gestational stage fetuses with excellent spatial resolution, tissue contrast, and diagnostic potential [6, 17–20].

As both UHF-MRI and micro-CT prove to be adequate modalities in visualizing different anatomical structures, the combination of both modalities may potentially be applied in future postmortem fetal imaging. When combining these modalities however, the effects of fixation and storage of specimens in formaldehyde solutions, such as paraformaldehyde (PFA), and of iodine-staining on UHF-MRI should be evaluated since these solutions appear to affect T1, T2, and T2* relaxation times and therefore signal intensity (SI) [21].

This study therefore aimed to (1) assess the feasibility of postmortem UHF-MRI in visualizing the forming cartilage and ligaments in different developmental stages, (2) determine the contrast ratio (CR) between several musculoskeletal structures in order to quantify this feasibility, and (3) evaluate the influence of different staining and storage protocols (*i.e.*, iodine and formaldehyde solutions). In this study, we focused on imaging of the human fetal upper extremity because it provides an excellent example of musculoskeletal development and represents an intricate anatomical region for assessing spatial resolution and differences in tissue contrast.

## Methods

The present experimental study included specimens from the Dutch fetal biobank, located at Amsterdam University Medical Centers (Amsterdam UMC), location AMC, from December 2018 until December 2020. Maternal and paternal informed consent for donation to the Dutch Fetal Biobank was obtained after decision-making and prior to the induction of labor. Ethical approval was granted via the accredited Medical Research Ethics Committee Amsterdam UMC (METC 127 2016_285,#B2017369).

### Specimen

We included human fetal upper extremities of fetuses with gestational ages ranging from 19 to 24 weeks from both sexes, without congenital defects or with a congenital defect (*e.g.*, chromosomal abnormality), provided that these defects did not concern the anatomy of the upper extremities. Exclusion criteria were fetal specimens with a congenital defect that caused macroscopic or potential upper extremity deformities. As the present study was within the scope of the biobank, no additional consent was required.

### Fixation

All specimens were fixed in freshly dissolved 4% (w/v) PFA in phosphate-buffered saline (PBS) (10 mM Na_2_HPO_4_/NaH_2_PO_4_ and 150 mM NaCl, pH 7.6) for 48 h on a shaking tray at 4 °C. After fixation, all specimens were stored in a 4% PFA solution in PBS at 4 °C, except for specimen 6, which was stored in 0.2% PFA in PBS at 4 °C. Specimens were scanned at different time points after fixation in order to assess the effect of storage time.

### Imaging

*Ex vivo* UHF-MRI at 7.0 T of all seven specimens was performed using an MR Solutions scanner (model MRS 7024, MR Solutions, Guildford, UK) and depending on the specimen’s size either with a 35-mm diameter quadrature volume coil or a 20-mm diameter mouse head coil. In order to enable high-resolution imaging and maintain the natural distal radioulnar joint congruity as much as possible, the upper limb was detached proximally from the elbow joint. The samples were stabilized within a Falcon tube and the wrist joint was positioned as neutral as possible. The tubes were filled with Fomblin (FenS, Goes, the Netherlands), which is an inert and hydrophobic perfluoropolyether-based lubricant that gives no signal on MRI and provides susceptibility matching to reduce field inhomogeneities.

The specimens were imaged with an exploratory fast low angle shot (FLASH) sequence, with the following technical parameters: field of view from 36 × 18 × 18 to 70 × 35 × 35 mm^3^, depending on the size of the specimen; echo time (TE) 7 ms or 12 ms; pulse repetition time (TR) from 20 to 50 ms; flip angle from 25 to 40°; spatial resolution from 0.070 to 0.137 mm; matrix size 512 × 256 × 256; number of excitations from 6 to 20; and acquisition time from 4 h 20 min to 7 h 15 min, depending on the number of excitations and TR. Scan parameters varied between specimens in order to obtain optimal image contrast for different specimen sizes. Additional factors, such as temperature during imaging and manufacturing origin of the fixative, which are known to be of influence in the imaging protocol of fetal upper extremities, were kept constant [22, 23].

### Storage and tissue staining

**Fig. 1:**
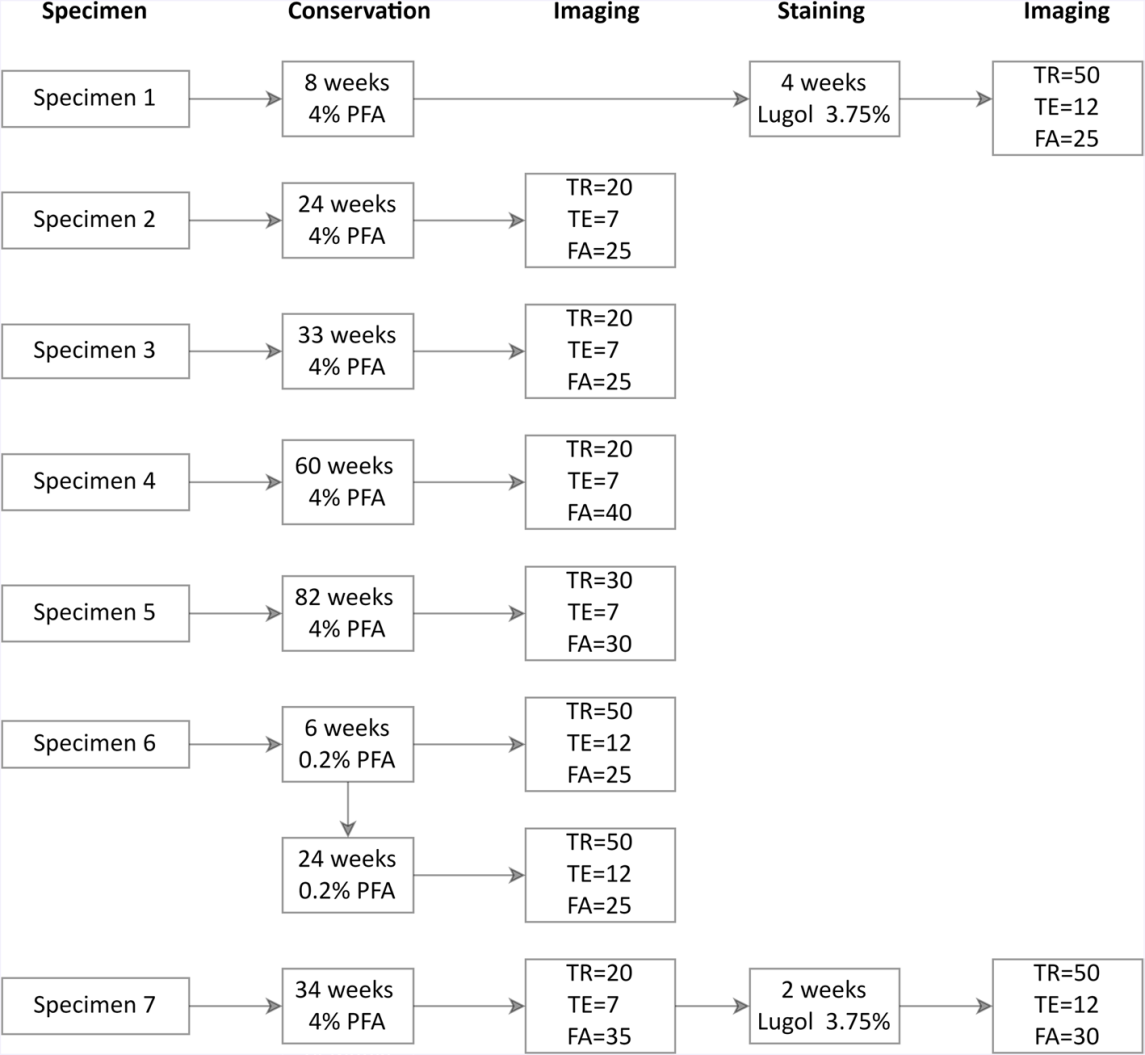
Diagram of ultra-high-field magnetic resonance imaging, staining, and fixation protocols for individual specimen. *FA* Flip angle, *PFA* Paraformaldehyde, *TE* Echo time, *TR* Repetition time

The effect of the following variables was assessed: (1) storage time, (2) PFA concentration used during storage, and (3) staining with Lugol’s solution. An overview of the different variables between specimens is provided in Fig. 1.

A decrease in pH level during staining induces tissue shrinkage [13]. As dissolved PFA in PBS can oxidize and form formic acid, we aimed to evaluate if storage in (relatively high percentage) 4% PFA might have a similar effect on MR image quality. Only one specimen was stored in 0.2% PFA as a reference. Since it was hypothesized that MR image quality is affected by storage in a higher percentage of PFA due to the possibility of acidification because of the formation of formic acid [13], we selected several storage times between 0 and 3 months in order to visualize a potential trend over time. After fixation in 4% PFA, specimens 1 to 5 were stored in 4% PFA and scanned at different periods of storage, *i.e.*, 8, 24, 33, 60, and 82 weeks, respectively, in order to assess the effect of storage time.

In order to compare the effect of storage percentage in 4% PFA to 0.2% PFA, one random specimen was stored in 0.2% PFA and imaged at two storage time points. Specimen 6 was stored in 0.2% (w/v) PFA after fixation and imaged after six weeks of storage to assess the effect of PFA concentration in comparison to specimen 1, which was stored in 4% PFA for a comparable duration. Thereafter, specimen 6 was imaged again at 24 weeks and then compared to specimen 2, which was stored in 4% PFA for the same duration.

For staining, a 3.75% Lugol’s solution was prepared from a 15% stock solution of Lugol (10 g KI and 5 g I_2_ dissolved in 100 mL bi-distilled water [6]. To evaluate the direct effect of staining, a specimen with relatively long conservation time, *i.e.*, 34 weeks in 4% PFA (specimen 7), was imaged prior and after 2 weeks of immersion in a 3.75% Lugol’s solution. Additionally, a specimen with a relatively short conservation time, *i.e.*, eight weeks in 4% PFA (specimen 1) was imaged after four weeks of immersion in 3.75% Lugol’s solution in order to evaluate a potential influence of storage time in combination with Lugol’s staining. Slight differences in staining time unfortunately occurred due to the dependence on MRI practicalities.

### Contrast ratios

In order to substantiate the visual soft-tissue contrast, two CRs between different types of soft tissue were determined: (1) the ratio of the signal intensity (SI) in the most homogenous chondrified carpal bone (CCB) from the proximal carpal row to the SI in the triangular fibrocartilage (TFC) and (2) the ratio of the SI of the CCB to the SI of the pronator quadratus muscle (PQM). The SIs that were used for the calculation of the CRs were determined by a single observer (G.S., physicist with 23 years of MRI experience) who was blinded for gestational age and fixation, storage, and staining protocol. The observer used a DICOM medical imaging viewer (Horos v.4.0.0, Horos project, Annapolis, MD, USA), in which a region of interest (ROI) was manually drawn within the visually most homogenous part in each structure in the mid-coronal slice. The mean SI, surface area, and standard deviation of each ROI were determined in Horos. The CRs were calculated by applying the following formulas where the SIs are the mean SIs within that ROI:

$${\mathrm{CR}}_{\mathrm{CCB}/\mathrm{TFC}}=\frac{\left({\mathrm{SI}}_{\mathrm{CCB}}-{\mathrm{SI}}_{\mathrm{TFC}}\right)}{({\mathrm{SI}}_{\mathrm{CCB}}+ {\mathrm{SI}}_{\mathrm{TFC}})}$$

$${\mathrm{CR}}_{\mathrm{CCB}/\mathrm{PQM}}=\frac{\left({\mathrm{SI}}_{\mathrm{CCB}}-{\mathrm{SI}}_{\mathrm{PQM}}\right)}{({\mathrm{SI}}_{\mathrm{CCB}}+ {\mathrm{SI}}_{\mathrm{PQM}})}$$

A positive CR indicates a relatively hyperintense SI of CCB compared to TFC or PQM, and a negative ratio indicates a relative hypointensity.

## Results

Seven human fetal upper extremities of six fetuses with gestational ages ranging from 19 to 24 weeks were included. An overview of the included fetal upper extremities is provided in Table 1.

**Table 1:** Specifications of fetal upper extremities

Specimen number	Gestational age (weeks)	Side	Reason for pregnancy termination
1^a^	21 + 1	Right	Hypoplastic left heart syndrome
2	19 + 0	Left	Social
3	20 + 4	Left	Holoprosencephaly
4	23 + 5	Left	Congenital heart disease
5^a^	21 + 1	Left	Hypoplastic left heart syndrome
6	22 + 4	Left	22q11 deletion
7	21 + 1	Left	Hypoplastic left heart syndrome

^a^Specimens 1 and 5 are the right and left upper extremities from the same specimen

### Feasibility and visualization of musculoskeletal structures

**Fig. 2:**
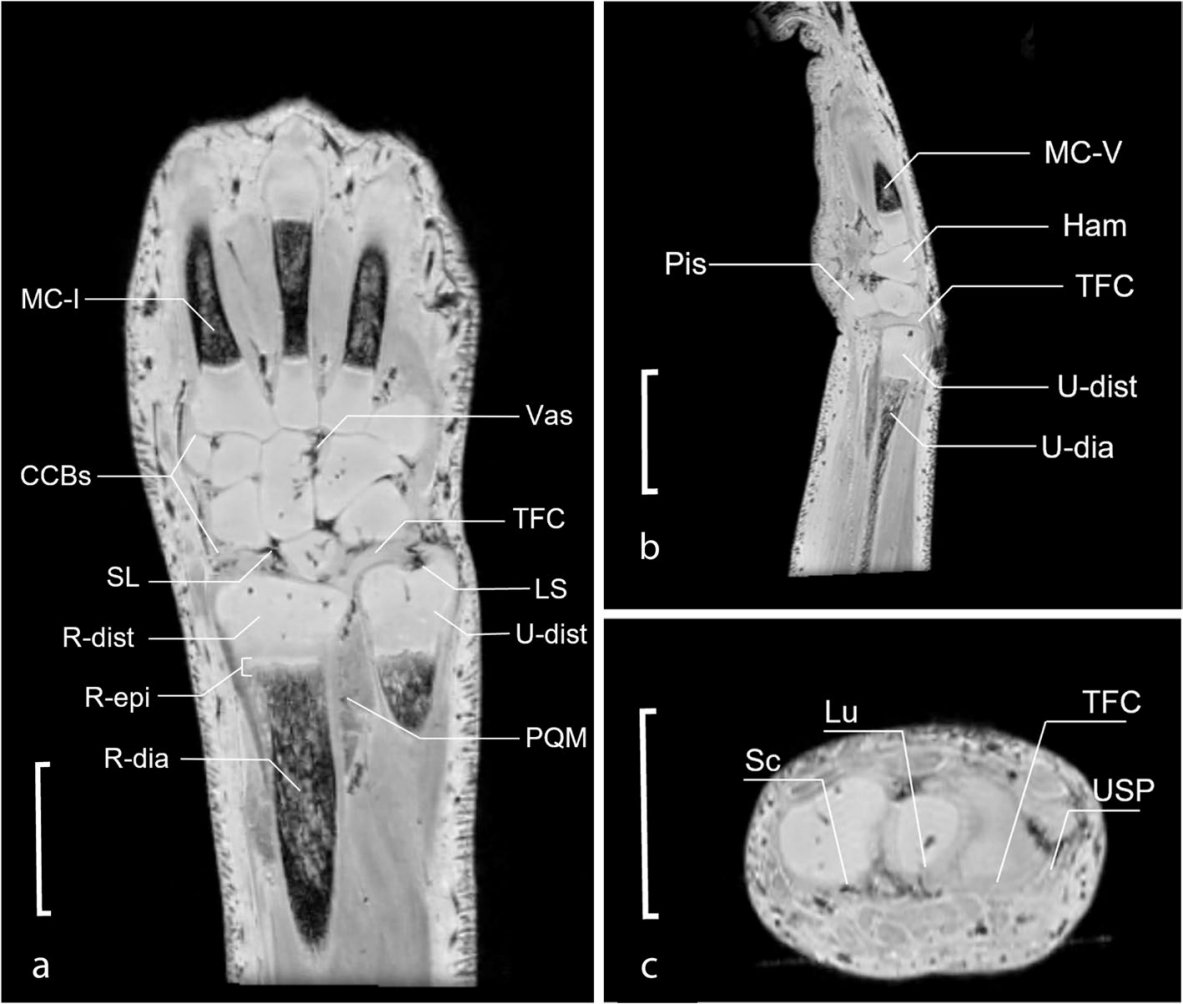
Magnetic resonance image of a mid-coronal T2*-weighted magnetic resonance image section of specimen 5 (**a**) with a multiplanar reconstruction in the sagittal (**b**) and axial plane (**c**). The scale represents 5 mm. *CCBs* Chondrified carpal bones, *Ham* Chondrified hamate bone, *LS* Ligamentum subcruentum intervening between proximal and distal lamina of TFC complex, *Lu* Chondrified lunate bone, *MC-1* Ossified diaphysis of the first metacarpal, *MC-V* Ossified diaphysis of fifth metacarpal, *PQM* Pronator quadrate muscle, *Sc* Chondrified scaphoid bone, *SL* Scapholunate ligament, *R-dia* Ossified radial diaphysis, *R-dist* Chondrified distal radius, *R-epi* Radial epiphyseal plate, *TFC* Triangular fibrocartilage, *U-dia* Ossified ulnar diaphysis, *U-dist* Chondrified distal ulna, *USP* Ulnar styloid process, *Vas* Penetrating vascular bundle

In Fig. 2, we show that the UHF-MRI protocol can be used to visualize and differentiate musculoskeletal structures due to the distinctive contrast between different soft tissues and the sharp morphological delineation of the structures within the upper extremity (specimen 1). Cartilage of the distal radius, the distal ulna, the carpal bones, and proximal metacarpals is clearly discernable from osseous, ligamentous, vascular, and muscular tissues. Note that the fibrocartilaginous tissue of the TFC is clearly distinguishable from the surrounding cartilaginous structures with the intervening vascular ligamentum subcruentum. Also, a distinct tri-lamination of physeal plates is discernable.

### Effect of storage time

**Fig. 3:**
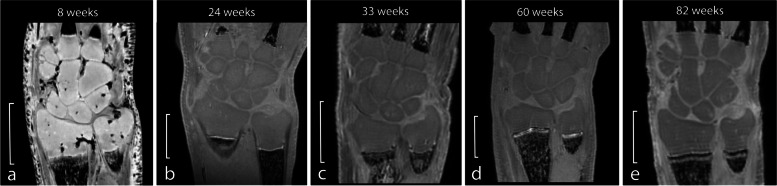
Magnetic resonance images of specimen 1 (**a**), specimen 2 (**b**), specimen 3 (**c**), specimen 4 (**d**), and specimen 5 (**e**), stored in 4% paraformaldehyde for different periods of time. The scale represents 5 mm

**Fig. 4:**
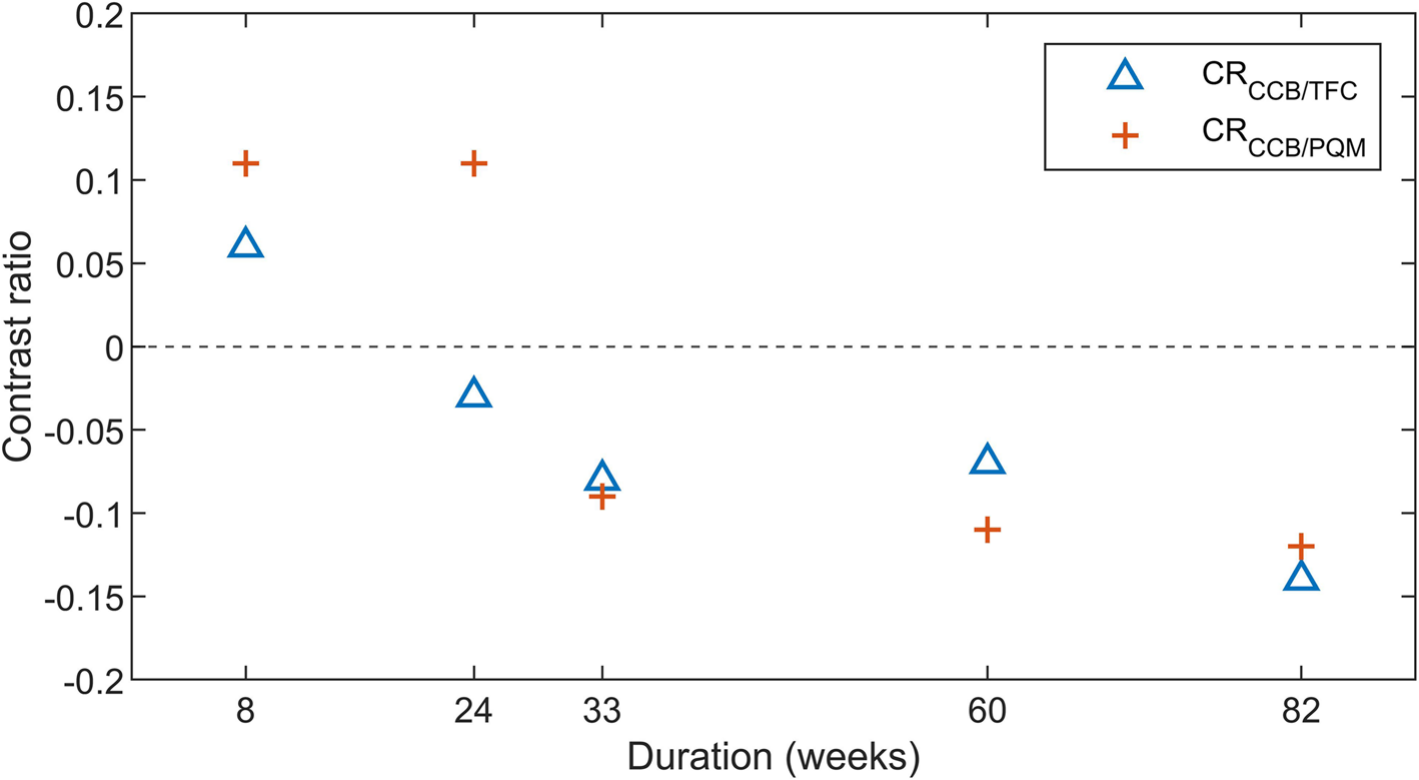
Contrast ratios of specimens 1, 2, 3, 4, and 5 plotted over time stored in 4% paraformaldehyde. *CCB* Chondrified carpal bone, *PQM* Pronator quadrate muscle, *TFC* Triangular fibrocartilage

Images of four specimens stored in 4% PFA for different time periods are shown in Fig. 3. Specimens were stored in 4% PFA for 8 weeks (specimen 1), 24 weeks (specimen 2), 33 weeks (specimen 3), 60 weeks (specimen 4), and 82 weeks (specimen 5) (Fig. 3a–e, respectively). Table 2 shows the ROI information and contrast ratios. Figure 4 presents the contrast ratios plotted over time. Contrast ratios were highest when storage time was lowest (8 weeks) with 0.06 for ${\mathrm{CR}}_{\mathrm{CCB}/\mathrm{TFC}}$ and 0.11 for ${\mathrm{CR}}_{\mathrm{CCB}/\mathrm{PQM}}$. At 24 and 33 weeks of storage, ${\mathrm{CR}}_{\mathrm{CCB}/\mathrm{TFC}}$ and ${\mathrm{CR}}_{\mathrm{CCB}/\mathrm{PQM}}$, respectively, resulted in negative values, indicating that the SI of CCB shifted into being hypointense compared to TFC and PQM. Further increase in storage times up to 82 weeks decreased contrast ratios to -0.14 for ${\mathrm{CR}}_{\mathrm{CCB}/\mathrm{TFC}}$ and -0.12 for ${\mathrm{CR}}_{\mathrm{CCB}/\mathrm{PQM}}$, accounting for a total decrease of 0.20 for ${\mathrm{CR}}_{\mathrm{CCB}/\mathrm{TFC}}$ and 0.24 for ${\mathrm{CR}}_{\mathrm{CCB}/\mathrm{PQM}}$ from initial ratios.

**Table 2:** Contrast ratios of specimens stored in 4% PFA for different amounts of weeks

Specimen number	Weeks in PFA	CCB			TFC			PQM			Ratios	
		Mean	SD	Area (mm^2^)	Mean	SD	Area (mm^2^)	Mean	SD	Area (mm^2^)	CCB/TFC	CCB/PQM
1	8	35,352.5	404.3	1.0	31,418.5	1,232.9	0.3	28,514.2	1,342.7	2.2	0.06	0.11
2	24	1,690.6	28.3	0.8	1,791.4	48.8	0.3	1,369.2	79.9	1.3	-0.03	0.11
3	33	1,541.2	28.9	1.3	1,821.3	67.9	0.3	1,848.5	64.0	2.1	-0.08	-0.09
4	60	1,378.0	30.0	1.6	1,586.6	48.9	0.9	1,730.2	32.4	4.4	-0.07	-0.11
5	82	1,535.4	63.7	1.0	2,024.3	73.6	0.5	1,958.2	67.7	3.4	-0.14	-0.12

*CCB* Chondrified carpal bone, *PFA* Paraformaldehyde, *SD* Standard deviation, *PQM* Pronator quadrate muscle, *TFC* Triangular fibrocartilage

### Effect of PFA concentration

**Fig. 5:**
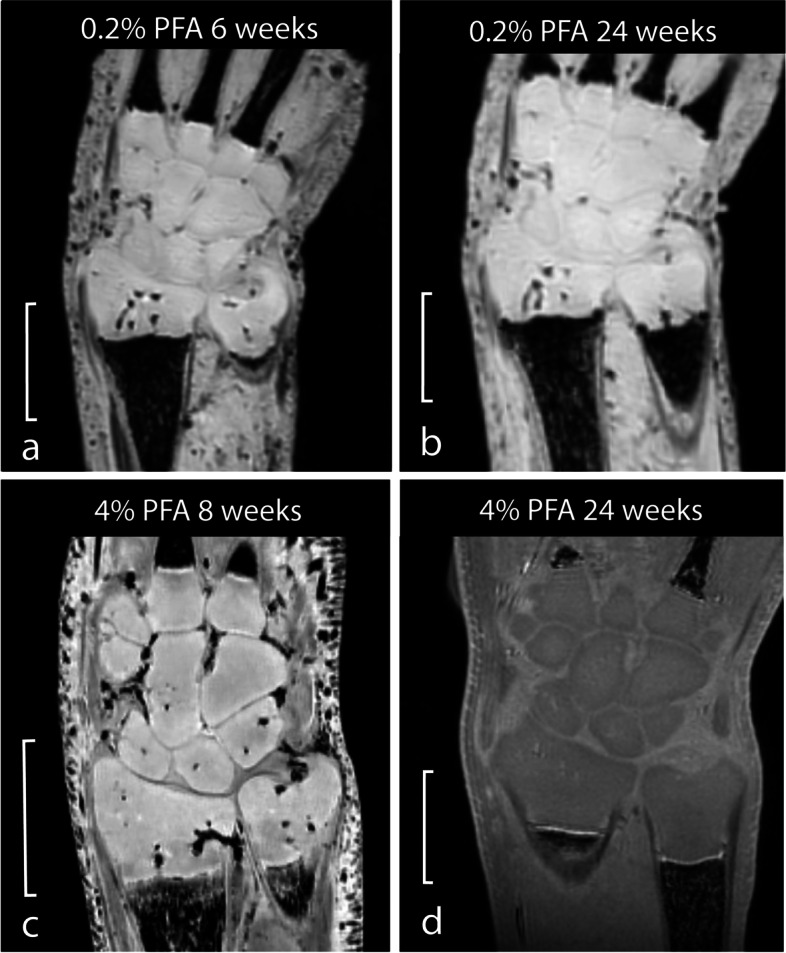
Magnetic resonance images of specimens stored in variations of paraformaldehyde concentrations and durations. Specimen 6 fixed in 4% for the first 24 h and then transferred to 0.2% paraformaldehyde (**a**, **b**). Specimens 1 and 2 are shown for comparison (**c**, **d**). The scale represents 5 mm

Images of the four specimens that were stored in different concentrations of PFA (*i.e.*, 0.2% and 4%) are shown in Fig. 5. The ROI information and contrast ratios are shown in Table 3. When comparing the CRs of the specimen stored in 0.2% PFA at 6 and 24 weeks, there is an increase of 0.04 for ${\mathrm{CR}}_{\mathrm{CCB}/\mathrm{TFC}}$. For the specimens stored in 4% PFA for comparable periods of time however, ${\mathrm{CR}}_{\mathrm{CCB}/\mathrm{TFC}}$ decreased with 0.09, creating a shift from a relatively hyperintense SI of CCB compared to TFC to hypointense SI. ${\mathrm{CR}}_{\mathrm{CCB}/\mathrm{PQM}}$ increased with 0.01 in 0.2% PFA, and ${\mathrm{CR}}_{\mathrm{CCB}/\mathrm{PQM}}$ did not differ between images over time in 4% PFA.

**Table 3:** Contrast ratios of specimens stored in different concentrations of PFA

Specimen number	PFA concentration	Weeks in PFA	CCB			TFC			PQM			Ratios	
			Mean	SD	Area (mm^2^)	Mean	SD	Area (mm^2^)	Mean	SD	Area (mm^2^)	CCB/TFC	CCB/PQM
6	0.2%	6	2,727.4	56.9	3.5	2,559.5	155.4	2.5	2,698.3	53.6	3.2	0.03	0.01
6^a^	0.2%	24	2,800.2	44.3	2.0	2,429.5	222.8	0.7	2,684.8	46.0	5.4	0.07	0.02
1	4%	8	35,352.5	404.3	1.0	31,418.5	1,232.9	0.3	28,514.2	1,342.7	2.2	0.06	0.11
2	4%	24	1,690.6	28.3	0.8	1,797.4	48.8	0.3	13,69.2	79.9	1.3	-0.03	0.11

*CCB* Chondrified carpal bone, *PFA* Paraformaldehyde, *PQM* Pronator quadrate muscle, *SD* Standard deviation, *TFC* Triangular fibrocartilage

^a^Second magnetic resonance image of specimen 6 performed with a different storage time

### Effect of staining

**Fig. 6:**
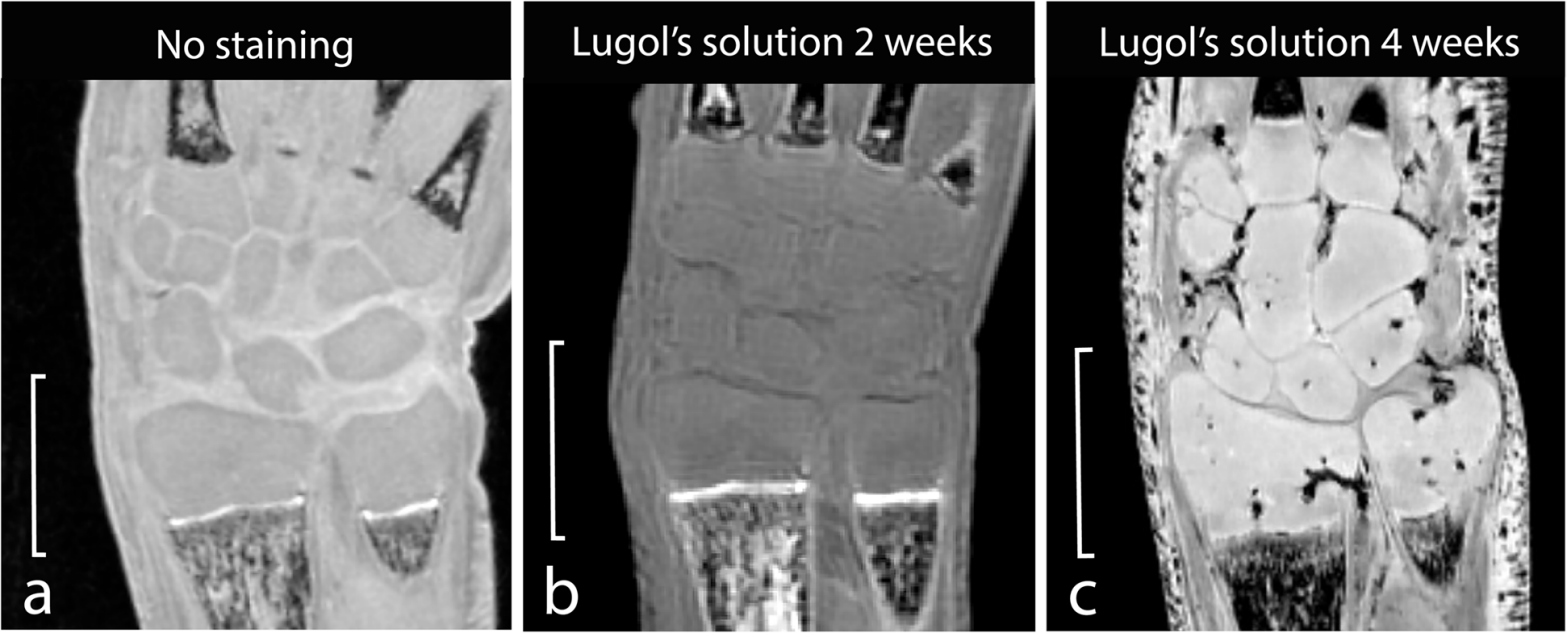
Magnetic resonance images of a specimen with and without staining with Lugol’s solution. Specimen 7 was imaged after storage in 4% paraformaldehyde for 34 weeks (**a**) and again after immersion in Lugol’s solution for 2 weeks (**b**). Specimen 1 was imaged after storage in 4% PFA for eight weeks and immersion in Lugol’s solution for four weeks (**c**). The scale represents 5 mm

To assess the effect of staining with Lugol’s solution on images made with UHF-MRI, one specimen which was stored in 4% PFA for 34 weeks was imaged prior and after 2 weeks of Lugol’s staining (specimen 7) and one specimen which was stored in 4% PFA for 8 weeks was imaged after 4 weeks of Lugol’s staining (specimen 1). The resulting contrast ratios and ROI information are shown in Table 4. After staining with Lugol’s solution for two weeks the CRs increased with 0.09 and 0.06 for ${\mathrm{CR}}_{\mathrm{CCB}/\mathrm{TFC}}$ and ${\mathrm{CR}}_{\mathrm{CCB}/\mathrm{PQM}}$, respectively. This increase indicated the shift of CCB from being relatively hypointense to relatively hyperintense compared to both PQM and TFC (Fig. 6). The CRs after staining for four weeks also indicated a relatively hyperintense CCB compared to both PQM and TFC, with CRs of 0.06 and 0.11 for ${\mathrm{CR}}_{\mathrm{CCB}/\mathrm{TFC}}$ and ${\mathrm{CR}}_{\mathrm{CCB}/\mathrm{PQM}}$, respectively (Fig. 6).

**Table 4:** Contrast ratios of specimens prior and after staining with Lugol’s solution

Specimen number	Lugol staining	CCB			TFC			PQM			Ratios	
		Mean	SD	Area (mm^2^)	Mean	SD	Area (mm^2^)	Mean	SD	Area (mm^2^)	CCB/TFC	CCB/PQM
7	No	1,779.0	50.3	1.3	2,088.1	100.5	0.7	1,811.4	49.3	2.3	-0.07	0.00
7^a^	2 weeks	1,211.6	34.5	1.0	1,158.6	66.1	0.6	1,079.7	61.4	1.9	0.02	0.06
1	4 weeks	35,352.5	404.3	1.0	31,418.5	1,232.9	0.3	28,514.2	1,342.7	2.2	0.06	0.11

*CCB* Chondrified carpal bone, *PFA* Paraformaldehyde, *PQM* Pronator quadrate muscle, *SD* Standard deviation, *TFC* Triangular fibrocartilage

^a^Second magnetic resonance image of specimen 7, performed after staining with Lugol’s solution

## Discussion

Embryological development is a 3D process, and due to its ability of visualizing different anatomical structures with high resolution, a combination of UHF-MRI and micro-CT can be expected in future embryological research. This is the first study that assessed the influence of storage time, storage solution concentration, and iodine staining on UHF-MRI of fetal specimens. The present study shows promising results regarding imaging soft tissue in the wrist joints of fetuses around 20 weeks of gestation imaged at 7 T with a FLASH sequence (TR 50 ms, TE 12 ms, and flip angle 25°). The images with the highest quality were obtained after storage in 4% PFA for a relatively short time period (eight weeks) after staining with Lugol’s solution 3.75% for two weeks. This affirms the feasibility of postmortem UHF-MRI as a tool to study cartilaginous and ligamentous anatomy development in human fetal extremities, even after prior Lugol staining for micro-CT imaging purposes. However, several storage and staining components appear to influence the discernibility of anatomical structures.

The only prior study on imaging the fetal upper extremities with UHF-MRI of which we are aware is by Langner et al. [8] who used a T2-weighted turbo spin-echo protocol on ten formalin-fixed upper extremities of fetuses ranging between eight and twelve weeks of gestation. This proof-of-principle study reported an interpolated in-plane pixel size of 20 μm with a slice thickness of 70 μm [8]. However, not enough sequence details were provided to calculate the real image resolution in order to compare this to the isotropic resolution of 70 μm that was reached in the present study. In general, we believe that isotropic voxels are superior to facilitate multi-planar reconstruction of fetal anatomy in all possible 3D orientations. Langner et al. [8] did show differentiation of forearm musculature with UHF-MRI at only eight weeks gestation and concluded that there was a direct correlation with conventional histology regarding chondrification. Yet, no soft-tissue contrasts or external factors of influence were reported.

Our observed effects of storage time in 4% PFA on CRs suggest that this factor influences tissue SIs and therefore the ability to discriminate between specific soft-tissue structures. Due to the changes in SIs, ${\mathrm{CR}}_{\mathrm{CCB}/\mathrm{TFC}}$ and ${\mathrm{CR}}_{\mathrm{CCB}/\mathrm{PQM}}$ both decreased with increasing storage time in 4% PFA. This is in accordance with changes in SIs that were described in studies on *ex vivo* adult human brain imaging where prolonged storage in formalin-based solution showed a decrease in T1 and T2 relaxation times [24, 25]. At 24 weeks in 4% PFA, an intensity shift even appears to occur in ${\mathrm{CR}}_{\mathrm{CCB}/\mathrm{TFC}}$, changing CCB SI from relatively hyperintense to relatively hypointense, which is visually supported (Fig. 3). This could be explained by a hypothetical stronger decrease in T1 relaxation times caused by PFA on TFC and muscular tissue than on chondrified osseous tissue, creating less T1 weighting and therefore higher SIs. On the other hand, a hypothetical stronger increase in T2* relaxation times of TFC and muscular tissue compared to chondrified osseous tissue or even a combination of these two relaxation properties could potentially explain this shift in CRs. These effects remain unexplored, yet the results support the suggestion by Langner et al. [8] that PFA storage time appears to be a factor that degrades differentiation between soft tissues.

Storage in 0.2% rather than 4% PFA barely changed contrast ratios between 6 and 24 weeks of storage, which was confirmed by visual inspection. If we compare CRs between storage in 0.2% PFA with 4% PFA at similar storage periods, there was a slight, yet visually supported decrease in the CR of especially CCB to TFC for 4% PFA after 24 weeks, which did not occur in a PFA solution of 0.2%. Hence, storage in 0.2% PFA would be preferable over 4% for storage for longer time periods. Additionally, the earlier change in ${\mathrm{CR}}_{\mathrm{CCB}/\mathrm{TFC}}$ (at 24 weeks) compared to ${\mathrm{CR}}_{\mathrm{CCB}/\mathrm{PQM}}$ (at 33 weeks) in 4% PFA is also remarkable. Since muscle fibers have shown increased penetration rates, earlier effects would be expected in ${\mathrm{CR}}_{\mathrm{CCB}/\mathrm{PQM}}$ than in ${\mathrm{CR}}_{\mathrm{CCB}/\mathrm{TFC}}$ [26]. Therefore, this indicates a difference in effect on relaxation properties between different soft tissues rather than differences in tissue penetration rates of PFA.

In micro-CT studies, it was reported that Lugol’s solution causes tissue shrinkage [27, 28]. As tissue shrinkage is accompanied by a reduction of water content, the magnetic susceptibility which is used for T2* weighting in FLASH sequences could also be affected, resulting in effects on tissue contrast [29]. The images prior and after staining with Lugol’s solution did show a small increase in the CRs, rather than the decrease that we found for PFA concentration and duration. This resulted in relatively hyperintense CCB after staining with Lugol’s solution, which was also supported visually (Fig. 6). As specimen 1 also had positive CRs, it might be possible that this effect was caused by Lugol staining. This change in CRs due to staining could be the result of various complex effects on T2* weighting of different tissues. Potentially, cross-linking and denaturation of proteins induced by fixation in PFA are influenced by pH levels and therefore indirectly affect T2* relaxation times. However, we did not measure pH changes and these variables remain to be explored in future studies. In any case, Lugol’s solution does not appear to cause artifacts or a decrease in soft-tissue contrast, which means that it can be safely used when preparing specimens for serial CT imaging and UHF-MRI.

The present study did have several limitations. In order to achieve sufficient resolution in a small and intricate anatomical region while maintaining enough signal-to-noise ratio for soft tissue, we chose to use a 3D-FLASH sequence with a relatively short TR and a matrix of 512 × 256 × 256, thereby reaching an isotropic resolution of up to 70 μm within the smallest wrist sample. The FLASH sequence has both T1 and T2* weighted contrast, which makes interpretation of the influence of staining on tissue contrast a complex one. The variation in MRI parameters (*i.e.*, TE, TR, and FA), however, is considered another major limitation that potentially obscured the effects of individual storage and staining parameters. We did not observe any indications that the variations substantially changed the SI trends that we observed, yet they might have influenced the SIs. We, therefore, recommend further research to consistently use a 3D FLASH protocol with a TR of 50 ms, TE of 12 ms, and flip angle of 25° and systematically vary storage and staining parameters to assess the exact effect on SIs.

Furthermore, we only focused on the imaging of tissue obtained from fetuses with gestational ages from 19 to 22 weeks. The feasibility of UHF-MRI as a modality for imaging specimens with lower and higher gestational ages therefore remains to be explored. Technical limitations such as resolution and bore size should however be anticipated in UHF-MRI of smaller and larger specimens, respectively. Future research could include fetal specimens with a wider range of gestation ages to explore the size limits.

Additionally, because of the limited availability of fetal tissue, it was not possible to vary all relevant parameters with respect to tissue preparation fully independently. Consequently, there was some overlap of the parameters for the different comparisons, and conclusions were based on a small amount of data points or even a single observation. We, therefore, stress that these results are preliminary and that future research should elaborate on the exact effects of Lugol’s solution prior to UHF-MRI. As this study is a preliminary assessment of different staining and storage protocols in UHF-MRI, a more prospective study with a larger sample size with a uniform MRI protocol and a single change in staining or storage variable per specimen is required.

In conclusion, our findings from this explorative study show that UHF-MRI is feasible for imaging musculoskeletal structures in the fetal upper extremities. PFA appears to decrease soft-tissue contrast, and therefore, we recommend storage in low concentrations (*i.e.*, 0.2%) and limitation of storage time in high concentrations (*i.e.*, 4%). Additionally, staining with Lugol’s solution, which is often performed for micro-CT imaging, does not appear to decrease and might even enhance soft-tissue contrast. As these findings are mainly preliminary, they need to be confirmed in prospective studies with larger sample sizes. We do conclude that UHF-MRI can be performed after contrast-enhanced micro-CT in order to fully exploit the advantages of both techniques.

## Data Availability

The datasets used and/or analyzed during the current study are available from the corresponding author on reasonable request.

## Abbreviations

CCB: Chondrified carpal bone
CR: Contrast ratio
FLASH: Fast low-angle shot
Micro-CT: Micro-computed tomography
PBS: Phosphate-buffered saline
PFA: Paraformaldehyde
PQM: Pronator quadratus muscle
ROI: Region of interest
SI: Signal intensity
TE: Echo time
TFC: Triangular fibrocartilage
TR: Repetition time
UHF-MRI: Ultra-high-field magnetic resonance imaging

## References

[1] Rodríguez-Niedenführ M, Burton GJ, Deu J, Sañudo JR (2001). Development of the arterial pattern in the upper limb of staged human embryos: normal development and anatomic variations. J Anat.

[2] Hita-Contreras F, Martinez-Amat A, Ortiz R (2012). Development and morphogenesis of human wrist joint during embryonic and early fetal period. J Anat.

[3] Al-Qattan MM, Yang Y, Kozin SH (2009). Embryology of the upper limb. J Hand Surg Am.

[4] Pichat J, Iglesias JE, Yousry T, Ourselin S, Modat M (2018). A survey of methods for 3D histology reconstruction. Med Image Anal.

[5] Gasser RF, Cork RJ, Stillwell BJ, McWilliams DT (2014). Rebirth of human embryology. Dev Dyn.

[6] Dawood Y, Strijkers GJ, Limpens J, Oostra RJ, de Bakker BS (2020). Novel imaging techniques to study postmortem human fetal anatomy: a systematic review on microfocus-CT and ultra-high-field MRI. Eur Radiol.

[7] Nijkamp JW, Sebire NJ, Bouman K, Korteweg FJ, Erwich J, Gordijn SJ (2017). Perinatal death investigations: what is current practice?. Semin Fetal Neonatal Med.

[8] Langner I, Stahnke T, Stachs O (2016). MR microscopy of the human fetal upper extremity - a proof-of-principle study. BMC Dev Biol.

[9] Okumura M, Ishikawa A, Aoyama T (2017). Cartilage formation in the pelvic skeleton during the embryonic and early-fetal period. PLoS One.

[10] Gabner S, Bock P, Fink D, Glosmann M, Handschuh S (2020) The visible skeleton 2.0: phenotyping of cartilage and bone in fixed vertebrate embryos and foetuses based on X-ray microCT. Development 147(11). 10.1242/dev.187633. 10.1242/dev.187633 32439754

[11] Lombardi CM, Zambelli V, Botta G (2014). Postmortem microcomputed tomography (micro-CT) of small fetuses and hearts. Ultrasound Obstet Gynecol.

[12] Arthurs OJ, Bevan C, Sebire NJ (2015) Less invasive investigation of perinatal death. BMJ. 351:h3598. 10.1136/bmj.h3598 10.1136/bmj.h3598 26155793

[13] Dawood Y, Hagoort J, Siadari BA (2021). Reducing soft-tissue shrinkage artefacts caused by staining with Lugol’s solution. Sci Rep.

[14] Trattnig S, Winalski CS, Marlovits S, Jurvelin JS, Welsch GH, Potter HG (2011). Magnetic resonance imaging of cartilage repair: a review. Cartilage.

[15] Tóth F, Nissi MJ, Zhang J (2013). Histological confirmation and biological significance of cartilage canals demonstrated using high field MRI in swine at predilection sites of osteochondrosis. J Orthop Res.

[16] Argentieri EC, Burge AJ, Potter HG (2018). Magnetic resonance imaging of articular cartilage within the knee. J Knee Surg.

[17] Shelmerdine SC, Hutchinson JC, Arthurs OJ, Sebire NJ (2020). Latest developments in post-mortem foetal imaging. Prenat Diagn.

[18] Thayyil S, Cleary JO, Sebire NJ (2009). Post-mortem examination of human fetuses: a comparison of whole-body high-field MRI at 9.4 T with conventional MRI and invasive autopsy. Lancet.

[19] Arthurs OJ, Guy A, Thayyil S (2016). Comparison of diagnostic performance for perinatal and paediatric post-mortem imaging: CT versus MRI. Eur Radiol.

[20] Staicu A, Albu C, Popa-Stanila R (2019). Potential clinical benefits and limitations of fetal virtopsy using high-field MRI at 7 Tesla versus stereomicroscopic autopsy to assess first trimester fetuses. Prenat Diagn.

[21] Ullmann JF, Cowin G, Kurniawan ND, Collin SP (2010). Magnetic resonance histology of the adult zebrafish brain: optimization of fixation and gadolinium contrast enhancement. NMR Biomed.

[22] Birkl C, Soellradl M, Toeglhofer AM (2018). Effects of concentration and vendor specific composition of formalin on postmortem MRI of the human brain. Magn Reson Med.

[23] Vučković I, Nayfeh T, Mishra PK (2020). Influence of water based embedding media composition on the relaxation properties of fixed tissue. Magn Reson Imaging.

[24] Shatil AS, Uddin MN, Matsuda KM, Figley CR (2018). Quantitative ex vivo MRI changes due to progressive formalin fixation in whole human brain specimens: longitudinal characterization of diffusion, relaxometry, and myelin water fraction measurements at 3T. Front Med (Lausanne).

[25] Dawe RJ, Bennett DA, Schneider JA, Vasireddi SK, Arfanakis K (2009). Postmortem MRI of human brain hemispheres: T2 relaxation times during formaldehyde fixation. Magn Reson Med.

[26] Thavarajah R, Mudimbaimannar VK, Elizabeth J, Rao UK, Ranganathan K (2012). Chemical and physical basics of routine formaldehyde fixation. J Oral Maxillofac Pathol.

[27] Heimel P, Swiadek NV, Slezak P (2019). Iodine-enhanced micro-CT imaging of soft tissue on the example of peripheral nerve regeneration. Contrast Media Mol Imaging.

[28] Degenhardt K, Wright AC, Horng D, Padmanabhan A, Epstein JA (2010). Rapid 3D phenotyping of cardiovascular development in mouse embryos by micro-CT with iodine staining. Circ Cardiovasc Imaging.

[29] Chavhan GB, Babyn PS, Thomas B, Shroff MM, Haacke EM (2009). Principles, techniques, and applications of T2*-based MR imaging and its special applications. Radiographics.

